# Generational Differences in Work-Family Conflict and Synergy

**DOI:** 10.3390/ijerph10062544

**Published:** 2013-06-19

**Authors:** Nicholas J. Beutell

**Affiliations:** Hagan School of Business, Iona College, 715 North Avenue, New Rochelle, NY 10801, USA; E-Mail: NBeutell@iona.edu; Tel.: +1-914-633-2663; Fax: +1-914-633-2012

**Keywords:** generational differences, work-family conflict, work-family synergy, mental health, self-rated health, job pressure, social support, autonomy, satisfaction

## Abstract

This paper examines differences in work-family conflict and synergy among the four generational groups represented in the contemporary workforce: Generation Y Generation X, Baby Boomers, and Matures using data from the *2008 National Study of the Changing Workforce* (*n* = 3,502). Significant generational differences were found for work-family conflict (work interfering with family and family interfering with work) but not for work-family synergy. Mental health and job pressure were the best predictors of work interfering with family conflict for each generational group. Work-family synergy presented a more complex picture. Work-family conflict and synergy were significantly related to job, marital, and life satisfaction. Implications and directions for future research are discussed.

## 1. Introduction

Managing workers from different generational groups has received increasing attention from managers and researchers. This interest has been fueled by the notion that generational groups differ with respect to their values, interests, motivations, and style of organizational adaptation. Understanding these potential differences might help in crafting organizational structures and programs to enable each group to be maximally productive (e.g., managing the work-family interface). Recent research by Callanan and Greenhaus [1], for example, discusses human resource and career issues facing the Baby Boom cohort, issued a “call to action” to organizations in this regard, while other investigators have argued for more research on generational effects [2]. The goal of this paper was to determine whether generational cohorts vary with respect to work-family conflict and synergy.

### 1.1. Generational Groups

Mannheim [3] is credited with developing the concept of generations as they are now conceived: as cohorts that share important life experiences that have a deep and lasting impact. As such, generational models attempt to explain the interaction between individuals and the historical events that both shape and are shaped by, the cohorts. As Scott [4] has noted: “Those born at the same time, may share similar formative experiences that coalesce into a “natural” view of the world. This natural view stays with the individual throughout their lives and is the anchor against which later experiences are interpreted. People are thus fixed in qualitatively different subjective areas”.

Eyerman and Turner [5] proposed a modification of Manheim’s concept as follows: “generation is defined as a cohort of persons passing through time who come to share a common habitus, hexis and culture, a function of which is to provided them with a collective that serves to integrate the cohort” (p. 91). This suggests that generational effects transcend age *per se*. Generation means being born at a certain time period within a specific zeitgeist that is shaped by major developmental events. As such, some of the “formative experiences” that shaped the four cohorts studied here have been discussed by previous researchers, notably:

Xers (29–43 years old in 2008, *n* = 992) and Boomers (44–62 years old in 2008, *n* = 1,830) represent the two largest cohorts in the U.S. workforce. Boomers, born between 1946 and 1964, represented the 17 million additional births following World War II. Xers were born roughly between 1963 and 1983. The “X” in Xers, according to Coupland [6], referred to the namelessness of the group, aware of its own existence, but overshadowed by the huge number of Boomers. Finally, Matures (63–83 years in 2008, *n* = 336), sometimes called the Silent Generation, were born between 1925 and 1945 and characterized as a group that suffered through war and economic depression ([7], p. 508).

GenY (below age 29 in 2008, *n* = 294) is the newest generational group (sometimes called millennials). One of the major factors influencing this cohort is technology and the internet [8]. Gen Y is characterized as having a strong desire for work/life balance, rapid career advancement, and higher levels of interest in international travel than other generational cohorts [9]. We need to develop a more complete understanding of generational cohorts in the workplace and what factors contribute to work-family conflict and synergy for each. 

Given the high level of interest in generational differences, few empirical studies (e.g., [7]) have investigated work-family conflict and synergy among generational cohorts: Generation Y (GenY), Generation X (Xers), Baby Boomers (Boomers), and Matures (note that studies have examined three of these groups but not Gen Y [7].) And, while Klun [10] used a case analysis to study work-life balance among Xers and GenY, this study did not specifically examine work-family conflict and synergy.

There have been a number of other studies that have looked at generational effects on careers [11,12,13,14], psychological contracts [15], job satisfaction and turnover intentions [16], human resource and workforce issues [17], and on work values, attitudes, beliefs, and expectations [8,12,18,19,20] And, while a recent meta-analytic review questioned the idea of generational differences in work-related attitudes [2], our goal is to expand previous work that has reported such differences for work and family domains [7].

This investigation was undertaken to address three research questions: Are there generational differences with respect to work-family conflict and synergy? What are the antecedents of work-family conflict and synergy for generational cohorts? Are work-family conflict and synergy correlated with satisfaction outcomes (*i.e.*, job, marriage, life) for each cohort? These questions are addressed using the 2008 *National Study of the Changing Workforce*.

### 1.2. Work-Family Conflict and Synergy

Greenhaus and Beutell [21] defined work-family conflict as “a form of interrole conflict in which the role pressures from the work and family domains are mutually incompatible. That is, participation in the work (family) role is made more difficult by virtue of participation in family (work) role” (p. 77). The conflict literature is extensive and continues to proliferate. And, it is generally acknowledged that directionality of role interference is important: work can interfere with family as well as family interfering with work [22]. Maertz and Boyar [23] offer a recent review and critique on this line of research.

Roles need not be is a constant state of tension and conflict as shown in recent theory and research [24,25]. Various labels have been attached to this phenomenon: work-family facilitation [26], positive spillover [27], positive balance, enrichment [25], and synergy [7,28]. Synergy appears to be the best fit for the variable measured here. Beutell [28] observed that: Work-family synergy refers specifically to positive energy and mood states that emerge from participating in work and family roles. And, distinct from related concepts, work-family synergy is conceptualized and measured as the frequency of experiencing positive energy and mood states as opposed to a discrete transfer between domains. As such, work-family synergy incorporates the temporal aspects of interaction between work and family roles (p. 651).

### 1.3. Generational Differences in Work and Family

The way that expectations are shaped for family and work roles can presumably be traced to formative experiences and expectations that are part of the zeitgeist for each cohort. (For the general historical factors affecting each group see [7,12,14]) Matures, for example, were raised when the modal family could be defined as traditional, an environment with fairly clear-cut norms and values including boundaries between work and family. Matures had experienced relatively traditional gender roles with the husband as breadwinner and the wife as homemaker. Boomers grew up in an era of shifting and blurring of gender roles (e.g., sexual liberation and the Women’s Movement). The vast majority of Boomers grew up in two-parent households although the increasing divorce rates portended change. These changes were fully felt by GenX who saw single-parent and blended families, working mothers, latch-key children (day care was not readily available), dual-career couples in an environment of corporate downsizing, and a clear shift away for the “traditional” family (Xers, see [29]), and finally, GenY, who saw their moms go to work and one parent leave the household before they graduated from high school [30]. GenYs report happy childhoods and a tendency to be closer to their mothers than their fathers [30]. Even when two parents are present, this is not the two-parent family experienced by Boomers. GenYs tend to be accepting of gay marriages as well as living together without being married [30]. Obviously the picture is complicated, but, nevertheless, each cohort faced quite different circumstances in their formative years as just noted. As such, we believe that there is sufficient evidence and some empirical results [7] to expect that these groups would differ on work-family conflict and work-family synergy.

### 1.4. Predictors of Work-Family Conflict and Synergy

Three classes of predictors were selected based on previous work-family research: Health-related [31], social support [32], and job-related [33]. Our strategy was to use established variables from the work-family literature to see if these variables predict conflict and synergy for each generational group. Previous generational research has provided evidence of the importance of these variables [7].

#### 1.4.1. Health-Related

The literature on self-rated and mental health and work-family conflict and synergy continues to grow (e.g., [24,31,34,35,36]. Grzywacz and Bass [35] concluded that “work-family conflict and facilitation must be considered separately, and that adult mental health is optimized when family to work facilitation is high and family to work and work to family conflict is low” (p. 248). Other investigators reported inconsistencies in health effects but they reported that depression is a significant variable [37]. Carlson *et al.* [38] reported that work-family conflict was related to both types of health but that only physical health was associated with work-family enrichment. Beutell and Wittig-Berman [7] reported significant results for mental health, and to a lesser extent, self-rated health predicting conflict and synergy for three generational cohorts. The accumulated findings do suggest the importance of health as a predictor of both conflict and synergy. As such, we predict that mental health (higher levers of symptomatic behavior) is directly associated with WIF and FIW but inversely associated with WFS. Similarly, self-rated health is inversely correlated with WIF and FIW but positively correlated with WFS.

#### 1.4.2. Social Support

Having support from one’s supervisor and coworkers can serve as a buffer from work demands as well as managing work and family. A recent meta-analytic review [32] found that supervisor support, particularly those supervisors who are sensitive to work and family demands, can mitigate work to family conflict. Many studies have found inverse relationships between social support and conflict (e.g., [39]). Besides the possibility of ameliorating conflict, a supportive supervisor or coworkers might also increase positive feelings like work-family synergy [31]. Thus, supervisor and coworker support inversely related to WIF and directly associated with work-family synergy. 

#### 1.4.3. Job-Related

We included job resources as well as job demands for our work-related predictors. This was based on the Job-Demands Resources model that stress is the product of job demands exceeding job resources [40]. Employees endeavor to maintain a balance between demands and resources to the extent possible. We selected job opportunities and autonomy as the resource factors. Learning opportunities suggest that employees are engaged in their work and actively seeking for ways to develop additional knowledge and skills. Autonomy indicates that employees have control over and discretion in carrying out their work assignments. On the other hand, work pressure is a job demand suggesting tasks exceed available time, that work is fast-paced, and that hard work may not be sufficient to keep up.

We argue that the job resources of autonomy and learning opportunities increase what Greenhaus and Powell [25] have termed “affective and instrumental” resources serving to enhance synergy and reduce conflict. Job pressure, as a salient work demand, would tend to increase conflict between work and family and detract from the synergies between these roles. Work stressors have already been shown to be inversely associated with WIF and FIW (e.g., [22]). Thus, we expect that job the job resources (autonomy and learning opportunities) will reduce conflict and increase synergy while the work demand of job pressure will increase conflict and reduce synergy.

### 1.5. Satisfaction Outcomes

Satisfaction is frequently assessed as an outcome variable of conflict and synergy [7,41]. The accumulated empirical evidence suggests inverse relationships with WIF and FIW [37] but positive relationships with WF-S [26,41]. We expect to observe these relationships for the generational groups being investigated. This study goes beyond previous work by including GenY and investigating satisfaction as an outcome variable rather than just comparing generational groups on levels of satisfaction.

## 2. Methods

### 2.1. Sample

The participants (*n* = 3,502) responded to the 2008 *National Study of the Changing Workforce* conducted by Harris Interactive using a questionnaire designed by the Families and Work Institute Public Use Files [42] . Sample eligibility was limited to people who (1) worked at a paid job or operated an income-producing business, (2) were 18 years or older, (3) were in the civilian labor force, (4) resided in the contiguous 48 states, and (5) lived in a non-institutional residence—*i.e.*, household—with a telephone. In households with more than one eligible person, one was randomly selected to be interviewed. Interviewers offered cash honoraria as an incentive for completing the extensive interview (see the documentation for a complete description of the incentive system used for the 2008 sample). With respect to type of employment, 53% worked for a private, for-profit company, 9% worked for a non-profit organization, 18% worked for a governmental agency, and 19% were self-employed. Considering gender of the participants, the sample had 1,867 men (53.3%) and 1,635 women (46.7%). The generational cohorts were classified as follows in Table 1.

This corresponds most closely to the model advanced by Lancaster and Stillman [43] except we use the term “GenY” rather than Millennials. The vast majority of the participants (88%) were interviewed in 2008.

**Table 1:** Ages for generational cohorts by year.

	2007	2008
GenY	<28 years	<29 years
Xers	28–42	29–43
Boomers	43–61	44–62
Matures	62+	63+

### 2.2. Measures

All measures were compiled by the Families and Work Institute for their 2008 study. Some items and scales can be traced to the Quality of Employment Survey [44] and the Families and Work Institute 1992, 1997, and 2002 [45] studies.

#### 2.2.1. Work-Family Conflict and Synergy

Work interfering with family (WIF; α = 0.86) was measured using five items (“I frequently have no energy to do things with my family because of my job”). Family interfering with work (FIW; α = 0.82) also had five items (“I don’t have enough time for my job because of my family”). Finally, work-family synergy (WF-S) consisted of four items (α = 0.70) (e.g., frequency of having more energy to do things with family because of my job; having more energy at work because of my family/personal life). 

#### 2.2.2. Index of Mental Health

The survey documentation includes the following description of the mental health scale: The index of mental health was derived through a principal components analysis of items measuring depression and stress (e.g., how often did you feel depressed or hopeless in the last month?). Respondents indicated how frequently they experienced minor health problems, sleep problems affecting job performance, feeling nervous or stressed, unable to control important things in life, feeling unable to overcome difficulties, and depression. A note accompanying the construction of the index indicated that one third of the national sample exhibited signs of depression predictive of clinical depression according to psychiatric screening criteria [42]. The scale came as a standardized score (included in the *2008 National Study of the Changing Workforce* Public Use Files) with higher scores indicating more mental health symptoms. Coefficient alpha for the entire 2008 sample was 0.82. 

#### 2.2.3. Self-Rated Health

Self-rated health was measured by a single item “how would you rate your current state of health” on a four-point scale (poor, fair, good, excellent). Higher scores indicate better health.

#### 2.2.4. Supervisory and Coworker Support

Supervisor support (α = 0.91) was measured using a 9-item scale (e.g., “My supervisor or manager is understanding when I talk about personal or family issues that affect my work”) with responses ranging from “strongly disagree” to “strongly agree”. The items were summed with a high score indicating more support. Similarly, coworker support (e.g., “I have the coworker support I need to do a good job”) was measured by three items with higher scores indicating more support (α = 0.76).

#### 2.2.5. Learning Opportunities, Autonomy, and Work Pressure

Learning opportunities (α = 0.79) was measured using six items (e.g., “My job lets me use my skills and abilities and my job requires that I keep learning new things”). The six items were factor analyzed using the principal components method with varimax rotation. Only one factor was extracted indicating that the items were unifactorial. Higher scores indicate more learning opportunities. Autonomy (α = 0.77) was measured by four items (e.g., I have the freedom to decide what I do on my job) with higher scores indicating more autonomy. A similar autonomy measure has been used as an antecedent of work-family conflict [46]. Job pressure (α = 0.51) was also measured using three items (e.g., I never have enough time to get everything done on the job). Higher scores indicate more work pressure.

#### 2.2.6. Job Satisfaction, Marital Satisfaction, and Life Satisfaction

Domain satisfaction was measured as follows: job satisfaction (α= 0.74) was measured using three items: how satisfied are you with your job, would you recommend your job, and would you take the same job again; family satisfaction and life satisfaction were measured using single-items assessing overall satisfaction with higher scores indicating higher levels of satisfaction.

## 3. Results

### 3.1. Demographic Variables by Generational Groups

Table 2 shows means, standard deviations, and sample sizes for each generational group. Note that each generational group had significant work (work hours) and family (presence of a child less than 18 years of age for at least six months of the year).

### 3.2. Differences in Work-Family Conflict and Synergy

Our initial question considered differences in work-family conflict and synergy by generational cohort. ANOVAs revealed significant effects for conflict: WIF (*F_(3,3422)_* = 31.67, *p* < 0.001) and FIW (*F_(3,3439)_* = 15.38, *p* < 0.001). The analysis for WFS, however, failed to attain significance (*F_(3,3443)_* < 1.00, ns). Using the LSD method (in order to minimize *Type I* errors) indicated that, for WIF, GenY was significantly lower than Xers but higher Matures, Xers were significantly higher than Boomers and Matures, Boomers were lower than Xers but higher than Matures, and Matures were significantly lower than all other groups. For FIW, GenY was higher than Boomers and Matures, Xers were higher than Boomers and Matures, Boomers were lower than GenY and Xers but higher than Matures, and Matures were significantly lower on FIW than all other groups.

**Table 2:** Demographic variables by generational group.

	GenY		GenX		Boomers		Matures	
	M or %	SD	M or %	SD	M or %	SD	M or %	SD
*Est. Fam. Income*	62.70	43.59	96.81	81.90	110.78	104.06	79.70	99.72
*Age in Years*	23.69	3.15	36.68	4.30	52.54	5.23	68.21	5.64
*Work Hours*	27.86	16.18	31.13	16.65	27.61	18.58	17.57	19.83
*Child < 18*								
Yes	35.4%		70.1%		29.7%		4.2%	
No	64.6%		29.9%		70.3%		95.8%	
*Gender*								
Female	50.7%		50.7%		53.7%		54.8%	
Male	49.3%		50.5%		46.3%		45.2%	
*Marital Status*								
Legally Married	30.7%		64.2%		64.6%		58.3%	
Other Arrangement	69.3%		35.8%		35.4%		41.7%	
*Education*								
Hi Sch. or Less	40.1%		20.7%		23.7%		23.2%	
Some College	35.1%		33.1%		29.2%		28.0%	
4-Yr. Degree +	24.8%		46.3%		47.2%		48.8%	

*Note.* Sample sizes: GenY = 294; GenX = 992; Boomers = 1,830; and Matures = 336. Gender: 1 = male; 2 = female. Est. Fam. Income = Estimated family income in 2008 in 1,000s of $US. Work hours refer to regularly scheduled hours at main job. Child < 18 refers to having a child under the age of 18 present for at least six months during the year. Marital status: 1 = legally married; 2 = all other arrangements. Education has three categories: 1 = high school, GED, or less; 2 = some college; and 3 = 4-year degree and beyond.

### 3.3. Predictors of Work-Family Conflict and Synergy

The predictors (*i.e.*, health, social support, and work-related) were each regressed on WIF, FIW, and WFS. For WIF, mental health (high scores indicate more symptoms) and job pressure were the best predictors for each generational group (see Table 3 for βs) revealing positive relationships as predicted. Self-rated health was significantly related to WIF for GenY only. Supervisory support was associated with diminished levels of WIF for all groups except GenY. Coworker support was significant for GenX and Boomers. Job challenge/learning opportunities and autonomy were not related to WIF.

FIW findings (Table 4) indicated that mental health was the strongest overall predictor. The only other significant finding revealed that coworker support was negatively associated with FIW for Boomers. The other predictions (self-rated health, supervisor support, job challenge/learning opportunities, job pressure, and autonomy) were not significant for this sample.

Finally, the WFS (Table 5) results showed that mental health was associated with synergy for Baby Boomers only while supervisor support was significant for Xers and Boomers. Learning opportunities were related to synergy for GenY and for Boomers. Autonomy predicted synergy for GenY and Xers.

**Table 3:** Regressions for work interfering with family (WIF) on predictor variables by generational group.

Predictor Variables	GenY	Xers	Boomers	Matures
Mental health	0.26 **	0.34 **	0.41 **	0.22 **
Self-rated health	−0.17 **	0.00	0.02	−0.14
Supervisor support	−0.00	−0.15 **	−0.15 **	−0.23 **
Coworker support	−0.13	−0.10 **	−0.11 **	−0.09
Learning opportunities	0.03	−0.02	0.03	−0.08
Job pressure	0.23 **	0.19 **	0.20 **	0.32 **
Autonomy	−0.14 **	−0.03	−0.01	0.03
*F*	20.74 ***	43.39 ***	86.15 ***	9.01 **
*R^2^*	0.31	0.30	0.35	0.38

*Note.* Standardized regression coefficients (β) are presented. * *p* < 0.05 ** *p* < 0.01 *** *p* < 0.001.

**Table 4:** Regressions for family interfering with work (FIW) on predictor variables by generational group.

Predictor Variables	GenY	Xers	Boomers	Matures
Mental health	0.42 **	0.34 **	0.43 **	0.44 **
Self-rated health	−0.04	−0.05	−0.04	−0.05
Supervisor support	0.08	−0.07	0.02	−0.20
Coworker support	−0.11	−0.04	−0.11 **	0.05
Learning opportunities	0.02	−0.10 *	0.09	0.06
Job pressure	0.08	−0.02	0.04	−0.03
Autonomy	−0.03	0.12 **	−0.01	−0.11
*F*	13.42 ***	19.98 ***	44.30 ***	6.61 **
*R^2^*	0.23	0.10	0.22	0.31

*Note.* Standardized regression coefficients (β) are presented. * *p* < 0.05 ** *p* < 0.01 *** *p* < 0.001.

### 3.4. Work-Family Conflict (Synergy) and Satisfaction Outcomes

Our third research question addressed work-family conflict and synergy in relation to three types of satisfaction: job, marital, and life. This goes beyond previous research that examined overall differences in satisfaction for Xers, Boomers, and Matures [7]. Recall that we predicted negative relationships between WIF (FIW) and satisfaction and positive relationships between WFS and satisfaction. As shown in the Table 6, 23 of 36 βs were statistically significant with all of the significant findings in the predicted directions. Note that WIF was related to job satisfaction for all generational groups while FIW was related, but less strongly, to job satisfaction for Xers and Boomers only. Also, WIF was not significantly related to marital satisfaction for any generational group but FIW was associated with marital satisfaction for all groups except Matures. Finally, note that WIF, FIW, and WFS were related to life satisfaction with the exception of GenY for synergy (*i.e.*, 9 out of 12 βs attained statistical significance). The evidence indicates a high degree of support for this question.

**Table 5:** Regressions of work-family synergy (WFS) on predictor variables by generational group.

Predictor Variables	GenY	Xers	Boomers	Matures
Mental health	−0.08	−0.02	−0.07 *	−0.02
Self-rated health	0.08	0.04	0.04	−0.15
Supervisor support	−0.07	0.15 **	0.13 **	0.13
Coworker support	0.06	0.05	0.05	0.16
Learning opportunities	0.17 *	0.05	0.14 **	0.04
Job pressure	−0.04	−0.05	−0.02	−0.02
Autonomy	0.15 *	0.11 *	0.04	0.08
*F*	7.74 ***	9.51 ***	16.15 ***	1.78
*R^2^*	0.15	0.09	0.10	0.11

*Note.* Standardized regression coefficients (β) are presented. * *p* < 0.05 ** *p* < 0.01 *** *p* < 0.001.

**Table 6:** Regressions for work-family conflict and synergy on satisfaction outcomes by generational group.

Outcome Variables	GenY	Xers	Boomers	Matures
*WIF*				
Job satisfaction	−0.19 **	−0.25 **	−0.24 **	−0.23 **
Marital satisfaction	−0.10	0.02	−0.09 *	−0.03
Life Satisfaction	−0.30 **	−0.18 **	−0.22 **	−0.20 *
F	14.56 ***	35.37 ***	87.12 ***	7.06 **
R^2^	0.21	0.13	0.17	0.12
*FIW*				
Job satisfaction	0.01	−0.15 **	−0.04	−0.14
Marital satisfaction	−0.39 **	−0.17 **	−0.17 **	−0.13
Life Satisfaction	−0.31 **	−0.08	−0.20 **	−0.23 **
F	21.64 ***	19.22 ***	20.48 ***	8.19 **
R^2^	0.27	0.08	0.11	0.13
*WFS*				
Job satisfaction	0.36 **	0.15 **	0.17 **	0.13
Marital satisfaction	0.01	0.07	0.13 **	−0.01
Life Satisfaction	0.10	0.18 **	0.11 **	0.20 *
*F*	11.65 ***	22.47 ***	22.56 ***	3.31 *
*R^2^*	0.17	0.09	0.09	0.06

*Note*. Standardized regression coefficients (β) are presented. * *p* < 0.05 ** *p* < 0.01 *** *p* < 0.001. WIF = work interfering with family, FIW = family interfering with work, and WFS = work-family synergy.

## 4. Discussion and Conclusions

Our results augment and expand those reported by Beutell and Wittig-Berman [7], the only previous study we could find that focused specifically on predictors and outcomes of work-family conflict and synergy for the major generational cohorts in the US workforce. This study included the GenY cohort, used the 2008 *National Study of the Changing Workforce*, and examined satisfaction outcomes for each generational group.

### 4.1. Generational Differences in Conflict and Synergy

Differences in work-family conflict and synergy were strongly supported for WIF and FIW but not for WFS. The findings on synergy were surprising given that previous research [7] reported strong, statistically significant increases in synergy for the three generational groups in their sample. Our results indicated decreases in synergy compared with the previously reported findings for 2002 for Boomers and Matures with Xers remaining at the 2002 level for 2008. Variability in samples probably accounts for some of the difference but there are other explanations as well. For example, synergy increased sharply between 1997 and 2002 [7] and may have reached a plateau (note that average synergy levels are higher than average conflict levels, an interesting and important finding in itself in an area that has been dominated by a conflict paradigm) and/or synergy might not be quite as prevalent in difficult economic times.

Our data show slight decreases in WIF for Boomers and Matures and a slight increase for Xers who reported the highest level of WIF (this is the first examination of GenY who are essentially the same as Boomers in the level of WIF). Xers are in their peak family years with simultaneous demands on the work front accounting for significantly higher levels of WIF than the other generational groups. In a similar vein, FIW increased for Xers and Matures but decreased slightly for Boomers. The family demands for Xers would possibly account for the increase but it is not clear why Matures would increase in FIW. This could reflect differing views on retirement (who works and how much), lifestyle issues, involvement with children or grandchildren, or, perhaps, relocating to a post-employment environment.

Although we have identified quantitative differences for WIF and FIW, the qualitative effects of conflict, how such conflicts are experienced by the generational cohorts, may provide insights into the way in which family and career stages are actually experienced by the participants. The findings on the antecedents of conflict and synergy, discussed below, can provide some insights in this area. Beutell and Wittig-Berman reported that each group in that study (Xers, Boomers, and Matures) manifested a decrease in WIF (*i.e.*, 2002 scores were significantly lower than 1997 scores) pointing to the possibility that workers are finding successful ways to manage work-family balance along with corporate work-family initiatives that may be utilized by increasing numbers of employees.

### 4.2. Predictors of Conflict and Synergy by Generational Group

Note that mental health was the strongest predictor of both WIF and FIW for each of the groups. The results, indicating that more mental health symptoms are associated with higher levels of WIF and FIW, strongly supported the hypotheses thus corroborating previous findings [7,34] that also used a national probability samples. In fact, Frone [34] found that “employees who reported experiencing work-family conflict often were 1.99–29.66 times more likely than were employees who reported no work-family conflict to experience a clinically significant mental health problem” (p. 888). Our findings support the growing importance of mental health in the work-family literature [31]. Self-rated health, however, was not significantly related to either WIF or FIW although significant negative relationships for FIW have been reported [7].

The findings on job pressure are consistent with numerous previous studies [22]. Job pressure, a work stressor emanating from the physical as well as psychological demands of work [47], would be expected to heighten conflict with family role demands [21]. Thus, it is not surprising the job pressure increases WIF (but not FIW) for each generational group. Job pressure was the second strongest predictor, following mental health, in the present study.

Supervisor support predicted WIF for all cohorts except GenY (Table 2) but was not significantly related to FIW for any cohort. As one investigator noted “the supervisor is the literal and figurative face of organizational support for employees” [48]). Thus, an understanding supervisor can provide the emotional support to help reduce potential conflicts with family life (e.g., [49]). This would appear to be particularly important for GenY although supervisory support and WIF were not significantly related in this study. It is possible that a segment of the GenY participants, who are delaying marriage and family, may decrease the salience of supervisor support until family role demands become more salient. Knowing the degree to which the organization is family-supportive may be a significant factor as well [50]. In fact, family-supportive supervisor behaviors appear to be linked to employee performance and attitudes [51].

The predictors of WFS were somewhat weaker than those previously reported [7]. Some of the notable findings indicated that supervisor support predicted synergy for Xers and Boomers, learning opportunities for GenY and Boomers, and autonomy for GenY and Xers. Clearly more work on the sources and consequences of work-family synergy is needed [25].

### 4.3. Satisfaction Outcomes by Generational Group

The satisfaction findings reveal Matures to be the most satisfied with job, family, and life. The Matures, or Silent Generation, are entering “retirement with a hip lifestyle and unprecedented affluence” ([29], p. 41). Thus, there appears to be a tendency for all types of satisfaction to increase by generational group (*i.e.*, age-related increases, [52]). Overall, we found that conflict and synergy were significantly related to each of the three types of satisfaction investigated with conflict being negatively related and synergy being positively related to satisfaction. 

### 4.4. Limitations and Implications for Research

As with all studies some cautions are in order. While our findings used data from a high-quality, professionally-conducted study, all information was gathered from one extensive interview. The effects of social desirability and common method may tend to inflate hypothesized relationships. We cannot rule out that similar study variables may have been the result of a common cause. Longitudinal analyses that trace the trajectories of generational cohorts over time would be particularly valuable. Studying cohorts can give short shrift to the individual variability that exists within each group. Differences between generations are confounded by changes associated with maturation and aging, experience, family/life stage, and career stage [18]. There may also be temporal effects in the measurement of conflict and synergy; we need to know more about such variables: are they sustained effects or transient episodes like mood states? (see [23] review in this regard). Finally, some variables consisted of only one item (e.g., marital satisfaction) and the reliability estimate for job pressure was low.

It should be pointed out that the concept of generational groups or cohorts has received some criticism. Perhaps the most direct, and stinging criticism for adherents to this concept, suggests that generational groups are just a proxy for age. In other words, the zeitgeist during the formative years of development tend to account for very little variance relative to one’s chronological age. As a recent special issue on generational differences in the *Journal of Managerial Psychology* has suggested:

*This core theoretical premise underpinning generational differences is not however without criticism. There are, for example, problems in determining the exact temporal point at which to segregate the various generations (and some differences between studies on this, although the default option seems to be the Strauss and Howe [53] typology). Nor can it be assumed that all members of any given generation will experience the same key sociocultural or socioeconomic events in the same way [54]; that is, independent of social class, gender, ethnicity, or national culture, for example [55] (p. 859).*

Although in fairness, most of these criticisms have focused on work values and HR issues, and not work-family conflict and synergy per se, the above criticism of the generational concept should be considered.

Our findings do add to the evidence reported previously demonstrating generational effects in work-family literature [7]. Many additional research opportunities exist. We need to know more about changes in conflict, synergy, and domain satisfaction over longer periods of time, how conflicts are managed, and the role of coping behavior. Understanding the transitions of GenY and Xers, two cohorts who appear to value work-family balance, can help us to understand emerging gender roles and possible friction over espoused organizational work-family programs and actual practices in use. Simply having family-friendly programs available will not be sufficient, particularly for GenY and Xers, who would expect programs to be available and useable [56,57]. This is one area where supervisor and coworker support would be critical. Finally, we need to understand the dynamics that transform shared experiences to form a “world view” [4] for generational cohorts regarding issues like career, success, family, happiness, *etc.* The dissertation by Lyons [8] is an excellent discussion of the theoretical and methodological issues that may serve as an important source in future studies of generational groups.
